# The Effect of DNA Extraction Methods on Observed Microbial Communities from Fibrous and Liquid Rumen Fractions of Dairy Cows

**DOI:** 10.3389/fmicb.2018.00092

**Published:** 2018-01-31

**Authors:** Jueeli D. Vaidya, Bartholomeus van den Bogert, Joan E. Edwards, Jos Boekhorst, Sanne van Gastelen, Edoardo Saccenti, Caroline M. Plugge, Hauke Smidt

**Affiliations:** ^1^Top Institute Food and Nutrition, Wageningen, Netherlands; ^2^Laboratory of Microbiology, Wageningen University and Research, Wageningen, Netherlands; ^3^NIZO Food Research BV, Ede, Netherlands; ^4^Animal Nutrition Group, Wageningen University and Research, Wageningen, Netherlands; ^5^Laboratory of Systems and Synthetic Biology, Wageningen University and Research, Wageningen, Netherlands

**Keywords:** DNA extraction methods, rumen fluid, fibrous content, bacteria, archaea, fungi, 454 pyrosequencing, qPCR

## Abstract

DNA based methods have been widely used to study the complexity of the rumen microbiota, and it is well known that the method of DNA extraction is a critical step in enabling accurate assessment of this complexity. Rumen fluid (RF) and fibrous content (FC) fractions differ substantially in terms of their physical nature and associated microorganisms. The aim of this study was therefore to assess the effect of four DNA extraction methods (RBB, PBB, FDSS, PQIAmini) differing in cell lysis and/or DNA recovery methods on the observed microbial diversity in RF and FC fractions using samples from four rumen cannulated dairy cows fed 100% grass silage (GS100), 67% GS and 33% maize silage (GS67MS33), 33% GS and 67% MS (GS33MS67), or 100% MS (MS100). An ANOVA statistical test was applied on DNA quality and yield measurements, and it was found that the DNA yield was significantly affected by extraction method (*p* < 0.001) and fraction (*p* < 0.001). The 260/280 ratio was not affected by extraction (*p* = 0.08) but was affected by fraction (*p* = 0.03). On the other hand, the 260/230 ratio was affected by extraction method (*p* < 0.001) but not affected by fraction (*p* = 0.8). However, all four extraction procedures yielded DNA suitable for further analysis of bacterial, archaeal and anaerobic fungal communities using quantitative PCR and pyrosequencing of relevant taxonomic markers. Redundancy analysis (RDA) of bacterial 16S rRNA gene sequence data at the family level showed that there was a significant effect of rumen fraction (*p* = 0.012), and that PBB (*p* = 0.012) and FDSS (*p* = 0.024) also significantly contributed to explaining the observed variation in bacterial community composition. Whilst the DNA extraction method affected the apparent bacterial community composition, no single extraction method could be concluded to be ineffective. No obvious effect of DNA extraction method on the anaerobic fungi or archaea was observed, although fraction effects were evident for both. In summary, the comprehensive assessment of observed communities of bacteria, archaea and anaerobic fungi described here provides insight into a rational basis for selecting an optimal methodology to obtain a representative picture of the rumen microbiota.

## Introduction

The bovine rumen is a complex microbial eco-system consisting of bacteria, archaea, protozoa and anaerobic fungi (Neocallimastigomycota). These microbes interact with each other to break down ruminant feed components, such as plant fibers. Bacteria are the predominant microorganisms in the rumen and hydrolyse feed-derived plant polysaccharides into short chain fatty acids (SCFAs), amino acids and gasses, namely H_2_ and CO_2_ (Russell and Hespell, 1981). The majority of the SCFAs are rapidly absorbed by the animal host for energy. Anaerobic fungi (Neocallimastigomycota) form a significant part of the rumen microbiota and play an important role in fiber digestion (Bauchop, 1979; Liggenstoffer et al., 2010; Gruninger et al., 2014). These anaerobic fungi were overlooked in early rumen studies due to their intimate association with the plant material during their extensive vegetative life cycle phase, with only the transient zoospores characteristic of their motile life cycle phase being detectable in the rumen fluid (RF) (Gruninger et al., 2014). Although ruminal methanogenic archaea cannot utilize dietary plant polysaccharides directly and comprise only approximately 0.3–3% of the total microbial biomass in the rumen, their functional relevance to rumen metabolism is significant. Archaea form methane (CH_4_) by utilizing CO_2_, H_2_, formate, and methanol, which are produced during fermentation of dietary material by other rumen microbes (Hungate, 1966b; Marvin-Sikkema et al., 1990; Teunissen et al., 1992). Methane is a potent greenhouse gas and represents a loss of dietary energy to the ruminant (Meale et al., 2012).

The study of rumen microbial diversity is essential for in-depth understanding of the complex microbial interactions that shape the rumen ecosystem. This understanding can then be used to beneficially improve ruminant productivity, whilst decreasing the environmental footprint of ruminant livestock production (Zhou et al., 2009). Previously, much of the pioneering work by Robert Hungate was performed using traditional microbiological methods, involving isolation and characterization of pure strains to assess the diversity and functionality of rumen microbial communities. These strains, however, represented only a relatively small proportion of the total rumen microbial diversity (Hungate, 1966a). The importance of using culture independent studies to allow identification of uncultured and novel taxa within the rumen microbiota was previously confirmed (Edwards et al., 2004; Creevey et al., 2014). Archaea which utilize the products from bacteria, are difficult to culture (Paul et al., 2012). For anaerobic fungi, only a limited number of the identified genera have been recovered in culture to date (Haitjema et al., 2014).

Although culture independent methods overcome some biases associated with culture dependent methods, they also introduce a new set of biases related to extraction and PCR. Several studies have shown that methods used to extract DNA from rumen-derived samples had a significant effect on the apparent microbial diversity observed using various different molecular techniques targeting the 16S ribosomal RNA (rRNA) gene. These techniques include single strand conformation polymorphism (SSCP), denaturing gradient gel electrophoresis (DGGE), quantitative PCR (qPCR) and next generation technology amplicon sequencing (Yu and Morrison, 2004; Henderson et al., 2013; Villegas-Rivera et al., 2013).

In terms of DNA extraction, RF and fibrous content (FC) fractions represent very different types of physical matrices for processing. A recent study by Henderson et al. (2013) showed that the bacterial communities associated with these two fractions differed from each other. For example, the predominant bacterial phyla in the rumen observed were Firmicutes and Bacteroidetes (Fouts et al., 2012), but the relative abundance of these phyla differed between the RF and FC fractions (Henderson et al., 2013). In the liquid fraction, the predominant bacterial community member was *Prevotella*, belonging to the Bacteroidetes phylum. In contrast, bacterial taxa belonging to the phyla Fibrobacteres and Firmicutes, particularly *Butyrivibrio, Succiniclasticum* and Lachnospiraceae, were relatively more abundant in the solid fraction. However, when the effects of different DNA extraction methods and two rumen digesta sampling methods were compared to each other, the choice of DNA extraction method affected the apparent microbial community structure significantly more than the sampling method (Henderson et al., 2013). Another study by Fliegerova et al. (2014) observed the clustering of microbial communities based on the type of RF processing (cheesecloth squeezed, centrifuged or filtered), storage conditions and DNA extraction method.

Differences in observed bacterial patterns due to extraction methods are often caused by the differences in cell lysis efficiency associated with the characteristic cell wall structure of Gram positive and Gram negative bacteria (Fliegerova et al., 2014). However, information on the biases associated with DNA extraction of the rumen FC relative to RF is limited. As primary fiber-degrading microbes are mainly attached to the dietary plant material (Dehority, 1991), it is important to assess the effect of DNA extraction methods on the observed FC and RF microbiota, and to what extent the extracts generated are reflective of the actual microbiota.

In this study, we evaluated the effect of four DNA extraction methods, that differ in cell lysis and/or DNA recovery procedures, on the outcome of microbiota compositional analysis of both RF and FC fractions. Description and discussion of the fraction effect was, therefore, also performed in order to place the DNA extraction method effects in context. Sample fractions were collected from four rumen cannulated dairy cows each fed different roughage-based diets that were previously shown to result in differences in methane emission (Van Gastelen et al., 2015). Quality and quantity of the extracted genomic DNA was evaluated prior to assessment of bacterial, archaeal and fungal communities with quantitative PCR (qPCR) and 454 based pyrosequencing of barcoded 16S rRNA gene and ITS PCR amplicons.

## Materials and Methods

### Animals and Diet

The samples used in this study were a subset of a larger study, of which the details have been described elsewhere (Van Gastelen et al., 2015). This study was conducted in accordance with Dutch law and approved by the Animal Care and Use Committee of Wageningen University and Research. Briefly, in the larger study 12 rumen cannulated cows were grouped into three blocks according to lactation stage, parity and milk production. The cows within each block were subsequently randomly assigned to one of four dietary treatments. All dietary treatments had a roughage to concentrate ratio of 80:20 based on dry matter. On a dry matter basis, the roughage consisted of either 100% grass silage (GS100), 67% GS and 33% maize silage (GS67MS33), 33% GS and 67% MS (GS33MS67), or 100% MS (MS100). One block of four cows was randomly selected from the above mentioned larger study to sample the RF and FC fractions in order to assess the effect of DNA extraction method on rumen microbiota analysis.

### Sample Collection, Preservation and Preparation

After 12 days of adaptation to the diet, the four rumen cannulated cows, i.e., one per dietary treatment, were sampled for RF and FC 3 h after morning feeding. RF was directly collected using a suction tube through the rumen fistula, and collected in 3 equal (∼200 ml) amounts from the front and middle of the ventral sac and from the cranial sac. After collection, the RF samples were pooled, thoroughly mixed, divided into aliquots of ∼50 ml, and immediately frozen on dry ice. The solid (fibrous) fraction was collected via the rumen cannula, and then firmly squeezed by hand. All samples were collected within a time span of 30 min, after which they were transported to the laboratory and stored at -80°C until DNA extraction. In order to facilitate DNA extraction in 2 ml lysis tubes, approximately 7.5 g of FC was ground using a mortar and pestle with liquid nitrogen, after which 0.2 g FC was weighed and used for extraction of DNA. RF samples were thawed, 1 ml aliquots centrifuged for 5 min at 9,000 × *g*, and the cell pellets used as the starting material for DNA extractions.

### DNA Extraction

Four different DNA extraction methods were compared in this study to represent different types and combinations of cell lysis mechanisms and/or DNA recovery procedures. All extractions were performed by one person. Each DNA extraction method was performed with eight samples, i.e., a RF and FC sample derived from four different cows, each of which were fed different diets. All extractions were performed once, with the exception of the sample from the cow fed the GS100 diet for which duplicate DNA extractions were performed. DNA extraction was performed using 0.2 g of ground FC or the cell pellet from 1 ml of RF.

#### Repeated Bead Beating (RBB)

Genomic DNA was extracted using the repeated bead beating plus column method, which was previously developed for bovine feces and rumen digesta (Yu and Morrison, 2004). Briefly, the prepared sample was mixed with 0.5 g of zirconium beads (0.1 mm; Biospec products), 4 glass beads (2.5 mm; Biospec products) and 1 ml of lysis buffer (500 mM NaCl, 50 mM Tris-HCl (pH 8), 50 mM EDTA, 4 % (w/v) SDS) in 2 ml lysis tubes with screw caps (BIOplastics BV) and then processed as the published protocol. The final genomic DNA was eluted in 100 μl AE buffer (10 mM Tris-HCl, 0.5 mM EDTA; pH 9.0).

#### Phenol Dependent Bead Beating (PBB)

Prepared samples were mixed with 940 μl TE buffer (10 mM Tris-HCl pH 7.6, 1 mM EDTA pH 8.0), followed by addition of 50 μl 10% (w/v) SDS and 10 μl Proteinase K (20 mg/ml), and then incubated at 55°C for 1 h. The mixture was then transferred to a 2 ml lysis tube containing 4 glass beads and 0.5 g zirconium beads (as used for the RBB protocol). Subsequently, 150 μl of buffered phenol (pH 7–8; Sigma–Aldrich) was added, followed by bead beating for 3 min using the bead beater (Precellys 24, Bertin technologies) at 5.5 m/s and cooled immediately on ice. The aqueous phase containing the nucleic acids was further mixed with 150 μl chloroform-isoamyl alcohol (24:1), and excess phenol was removed through centrifugation at 14,000 × *g* for 10 min at 4°C. The upper aqueous phase was removed and transferred to a new tube. The extraction with buffered phenol and chloroform-isoamyl alcohol was repeated. The nucleic acids were then precipitated from the combined aqueous fractions by adding 0.1 volume of 3M sodium acetate and 1 volume of isopropanol, and incubating at 4°C for 30 min followed by centrifugation. The pellets were washed once with 70% (v/v) ethanol and allowed to air-dry before being rehydrated in 100 μl of TE buffer.

#### Fast SPIN DNA Kit for Soil (FDSS)

Genomic DNA extraction was performed using the FastDNA SPIN kit for soil (MP Bio medicals, Solon, OH, United States) following the manufacturer’s instructions. Cell lysis in this kit was performed with sodium phosphate buffer and MT buffer in Lysing matrix E tube using the Precellys 24 bead beater for 40 s at a speed of 6.0 m/s, and the DNA purification was done using a binding matrix. DNA was eluted in 50 μl of DES (DNase/Pyrogen free water) that was provided with the kit.

#### PQIAmini

Genomic DNA was extracted following the method described by Zoetendal et al. (2006) with minor modifications (Van den Bogert et al., 2013). Briefly, prepared samples were mixed with 500 μl of TE buffer, and the genomic DNA was extracted from the re-suspended sample according to the Macaloid-based DNA isolation protocol with the use of Phase Lock Gel heavy tubes (5 Prime GmbH) and phenol during the phase separation step. To remove contaminating RNA, 250 μl of the aqueous phase was pre-treated with 3 μl RNAse A (10 mg/ml; QIAGEN GmbH) at 37°C for 15 min. Subsequent steps employed a modified version of the QIAamp DNA Stool Mini Kit (QIAGEN) protocol (Leimena et al., 2013). Initially, 22.5 μl proteinase K (20 mg/ml; Ambion) and 300 μl buffer AL from the QIAmp kit were added to the DNA extract followed by incubation at 70°C for 10 min. The rest of the protocol was performed following the protocol guidelines. DNA was finally eluted in 30 μl of nuclease free water.

#### Quality Control of DNA Extracts

The quality and quantity of the DNA was assessed using a Nanodrop ND-1000 spectrophotometer (NanoDrop^®^ Technologies). The integrity of the DNA was visualized using agarose gel electrophoresis with a 1% (w/v) agarose gel containing 1x SYBR^®^ Safe DNA gel stain (Invitrogen).

### qPCR Analysis

DNA extracted from RF and FC samples was used for quantification of bacteria, archaea and anaerobic fungi by qPCR. The amplification of bacterial and archaeal 16S rRNA genes, and anaerobic fungal 5.8S rRNA genes was performed in a BioRad CFX96 system (Bio-Rad Laboratories). All qPCR reactions were performed in triplicate. The resulting qPCR data was then processed, and principal component analysis (PCA) was performed using Canoco 5.0 (Ter Braak and Smilauer, 2012). The R software (version 3.0.2) was used for plotting and visualization purposes.

#### Bacteria and Archaea qPCR

To quantify bacterial 16S rRNA genes, the forward and reverse qPCR primers BAC 1369F (5′-CGGTGAATACGTTCYCGG-3′) and PROK 1492R (5′-GGWTACCTTGTTACGACTT-3′) were used (Suzuki et al., 2001). Archaeal 16S rRNA gene copies were quantified using primers 787F (5′-ATTAGATACCCSBGTAGTCC-3′) and 1059R (5′-GCCATGCACCWCCTC-3′) (Yu et al., 2005). The reproducibility of the bacterial qPCR assay (primers BAC 1369F and PROK 1492R) has been recently successfully confirmed for RF samples in our lab (Van Lingen et al., 2017). The reproducibility of the archaeal qPCR assay (primers 787F and 1059R) has been shown in a study focusing on bioreactor performance from methanogenic communities in microbial electrolysis cells (Lu et al., 2012), and the archaeal primers have been tested for their coverage by Yu et al. (2005). For bacteria and archaea the qPCR reaction mixture (25 μl) contained 12.5 μl 2X iTaq Universal SYBR Green Supermix (Bio-Rad Laboratories), 200 nM forward primer, 200 nM reverse primer, 10.5 μl nuclease free water, and 1 μl of 0.2 ng/μl (for bacteria) or 2 ng/μl template DNA (for archaea). The thermal cycling conditions for the bacterial and archaeal primer pairs included a pre-denaturing step at 95°C for 10 min, followed by 35 cycles of 95°C for 20 s, annealing at 56.3°C (for bacteria) or 60°C (for archaea) for 30 s and extension at 72°C for 30 s. The fluorescent products were detected at the last step of each cycle. Following amplification, melting temperature analysis of PCR products was performed to determine the specificity of the PCR. The melting curves were obtained by slow heating at 0.5°C/s increments from 60 to 95°C, with continuous fluorescence collection.

#### Anaerobic Fungi qPCR

The quantification of ruminal anaerobic fungi was carried out using the Neocallimastigales probe-based qPCR assay as previously described (Edwards et al., 2008). Briefly, primers Neo qPCR For (5′ TTG ACA ATG GAT CTC TTG GTT CTC 3′) and Neo qPCR Rev (5′ GTG CAA TAT GCG TTC GAA GAT T 3′) primers were used, targeting a conserved region (110 bp) of the 5.8S rRNA gene, along with a TaqMan probe (Neo: 5′ FAM-CAA AAT GCG ATA AGT ART GTG AAT TGC AGA ATA CG –TAMRA-3′). The reaction mixture (25 μl) contained 1 × TaqMan Universal PCR Probe Mix (Applied Biosystems), 750 nM of each primer, 200 nM of the probe and 1 μl of 2 ng/μl template DNA. The thermal cycling program was 50°C for 2 min, 95°C for 10 min (initial denaturation), followed by 40 cycles of 95°C for 15 s (denaturation) and 60°C for 1 min (primer annealing and extension). At the end of each cycle, the accumulation of PCR products was detected by monitoring the fluorescence signal from the probe.

#### Standard Curve Preparation

Standard curves were generated using purified PCR products as a template. The bacterial 16S rRNA gene PCR product was obtained with universal bacterial primers 27F and 1492R (Suzuki et al., 2000), using DNA extracted from *Ruminococcus albus* SY3 [kindly provided by Prof. R. John Wallace from the Rowett Research Institute (now part of the University of Aberdeen)]. The archaeal 16S rRNA gene PCR product was obtained with universal archaeal primers 25F and 1492R (Dojka et al., 1998; Suzuki et al., 2000), using DNA extracted from *Methanosarcina mazei* MC3 (DSM-2907). The anaerobic fungal 5.8S rRNA gene PCR product was obtained with the Neo qPCR Rev and Neo qPCR Rev primers using DNA extracted from a FC sample from the cow which was fed GS100. All the PCR products were purified with a Purelink PCR Purification kit (Invitrogen), with high-cut off binding buffer B3, and the concentration was measured using Nanodrop. The DNA concentration and amplicon size was used to calculate the number of amplicon copies, and then 10-fold serial dilutions in water were made from 10^8^ to 10^2^ amplicon copies/μl.

### Amplification of Target Regions for Pyrosequencing

#### Bacterial Community Assessment

Bacterial community composition was assessed as described previously (Van den Bogert et al., 2013). Briefly, a PCR was performed to obtain barcoded amplicons from the V1-V2 region of the 16S rRNA gene, using the 27F-DegS forward primer (5′-GTTYGATYMTGGCTCAG-3′) (Van den Bogert et al., 2011) appended with the pyrosequencing titanium sequencing adapter A (5′-CCATCTCATCCCTGCGTGTCTCCGACTCAG-3) and an 8 nt sample specific barcode (Hamady et al., 2008) and an equimolar mix of two reverse primers 338R I – (5′-GCWGCCTCCCGTAGGAGT-3′) and 338R II – (5′-GCWGCCACCCGTAGGTGT-3′) that were appended with the pyrosequencing titanium adapter B (5′-CCTATCCCCTGTGTGCCTTGGCAGTCTCAG-3′) at the 5′ end (Guss et al., 2011). The reverse primers are based on three previously published EUB 338 probes (Daims et al., 1999). PCRs were performed using a thermocycler (G storm) in a total volume of 100 μl containing 20 μl 5 × HF buffer (Finnzymes), 2 μl PCR Grade Nucleotide Mix (2 mM each), 2 units Phusion^®^ Hot Start II High Fidelity DNA polymerase (Finnzymes), 500 nM of both the barcoded forward and reverse primer, 65 μl nuclease free water and 2 μl of 20 ng/μl template DNA. The PCR program consisted of an initial denaturation step at 98°C for 30 s, followed by 30 cycles of 98°C for 10 s, 56°C for 20 s and 72°C for 20 s, with a final extension step at 72°C for 10 min. Expected PCR product size (311 bp) was confirmed by agarose gel electrophoresis using 5 μl of PCR product on a 1% (w/v) agarose gel containing 1x SYBR^®^ Safe. Non-template negative control PCR reactions were performed alongside each PCR amplification, and were confirmed to yield no product. PCR products were purified with the High PCR Pure Clean-up Micro kit (Roche) followed by quantification using the Qubit dsDNA BR assay kit (Invitrogen). Purified PCR products were mixed in equimolar amounts (400 ng per sample), and the pooled amplicons were purified using a DNA gel extraction kit (Millipore) according to manufacturer’s guidelines. The pooled amplicons were then quantified using the Qubit dsDNA BR assay kit, and the sequences determined with a 454 Life Sciences GS-FLX platform using Titanium sequencing chemistry (GATC-Biotech, Konstanz, Germany).

#### Archaeal Community Assessment

A method adapted from Jaeggi et al. (2014) was used for archaeal composition analysis. Briefly, barcoded amplicons of 16S rRNA genes were generated by PCR using the 340F forward primer (5′-CCCTAYGGGGYGCASCAG-3′) (Gantner et al., 2011) that was 5′-extended with the titanium adaptor A and an 8 nt sample specific barcode, and the 1000R reverse primer [5′-GGCCATGCACYWCYTCTC-3′ (Gantner et al., 2011)] that was appended with the titanium adaptor B at the 5′-end. PCRs were performed in a total volume of 50 μl containing 20 ng of template DNA, 200 nM of each of the forward and reverse primer, 1 U KOD Hot Start DNA Polymerase (Novagen), 5 μl KOD-buffer (10×), 3 μl MgSO_4_ (25 mM), 5 μl dNTP mix (2 mM each), and 33 μl nuclease free water. PCR conditions were: initial denaturation step at 98°C for 30 s followed by 25 cycles of 98°C for 10 s, 52°C for 20 s, and 72°C for 20 s, and a final extension step of 72°C for 10 min. PCR product size (660 bp) was confirmed by agarose gel electrophoresis using 5 μl of PCR product on a 1% (w/v) agarose gel containing 1x SYBR Safe. Non-template negative control PCR reactions were performed alongside each PCR amplification and were confirmed to yield no product. The PCR amplicon (∼660 bp) was subsequently purified using the MSB Spin PCRapace kit (Invitek), and the concentration was determined using the Qubit dsDNA BR assay kit. Purified PCR products were mixed in equimolar amounts by pooling 200 ng of the purified PCR products of each sample. The pooled sample was purified using the Purelink PCR Purification kit, with high-cut off binding buffer B3, and pyrosequenced on the 454 Life Sciences GS-FLX platform using Titanium sequencing chemistry (GATC-Biotech, Konstanz, Germany).

#### Fungal Community Assessment

PCR was performed to obtain barcoded amplicons from the fungal ITS1 region, using the ITS1FA.001 (5′-CTTGGTCATTTAGAGGAAGTAA-3′) forward primer appended at the 5′-end with titanium sequencing adapter B and the reverse primer (5′-TCCTCCGCTTATTGATATGC-3′) appended with titanium sequencing adapter A and a 6 nt sample specific barcode. PCRs were performed using a thermocycler (Biometra) in a total volume of 50 μl containing 5 μl 10x KOD buffer, 5 μl dNTP mix (2 mM each), 3 μl MgSO_4_ (25 mM), 1 μl KOD polymerase, 400 nM of both the forward and the reverse primer, nuclease free water, and 20–50 ng of template DNA. The PCR program consisted of an initial denaturation step at 95°C for 2 min, followed by 35 cycles of 95°C for 20 s, 51°C for 10 s, and elongation at 70°C for 15 s, with a final extension step at 70°C for 5 min. Expected PCR product size (variable between 350 and 750 bp) was confirmed by agarose gel electrophoresis using 5 μl of PCR product on a 1% (w/v) agarose gel containing ethidium bromide. Non-template negative control PCR reactions were performed alongside each PCR amplification, and were confirmed to yield no product. PCR products were purified with MSB spin PCRapace kit followed by quantification using Nanodrop. Purified PCR products were mixed in equimolar amounts (200 ng per sample), and the pooled amplicons were purified using MSB spin PCRapace kit according to manufacturer’s guidelines. The pooled amplicons were then quantified by Nanodrop and pyrosequenced on the 454 Life Sciences GS-FLX platform using Titanium sequencing chemistry (GATC-Biotech, Germany).

### Pyrosequencing Data Analysis

The pyrosequencing data analysis for bacteria and archaea was carried out with a workflow employing the Quantitative Insights Into Microbial Ecology (QIIME) pipeline (Caporaso et al., 2010) using settings as recommended in the QIIME 1.2 tutorial. De-multiplexing and initial sequence quality filtering were done with the “split_libraries.py” script provided by QIIME using the default settings. OTU picking, alignment and taxonomic classification were done using the workflow script “pick_otus_through_otu_table.py” provided by QIIME using the default settings. Reads were filtered for chimeric sequences using Chimera Slayer (Haas et al., 2011), and clustering of Operational Taxonomic Units (OTUs) was performed with a similarity threshold of 97%. Additional data handling was done using in-house developed Python and Perl scripts. Taxonomic classification of bacteria and archaea was done using Ribosomal Database Project (RDP) classifier version 2.2 (Wang et al., 2007) using the database GreenGenes set gg_97_otus_6oct2010 as provided with QIIME 1.2. In order to obtain the most likely genus-level identification, sequences were compared to the corresponding RDP reference set using NCBI BLAST (Altschul et al., 1990). Data analysis for fungi was done using a workflow based on QIIME 1.8, using the BLAST method for taxonomic classification of ITS reads against the UNITE database (Abarenkov et al., 2010), using the training set of 07-04-2014. Shannon’s index and Chao1 richness index were calculated as implemented in QIIME using bacterial OTU-level data. Principal coordinate analysis (PCoA) analysis of weighted and unweighted UniFrac distances between samples was performed using QIIME with both the bacterial and archaeal OTU-level data. Redundancy analysis (RDA) was performed using Canoco 5 (Smilauer and Leps, 2014) to assess the relationship between family level like phylogenetic groupings of OTU and DNA extraction methods or rumen fractions. The raw sequence data for the bacterial, archaeal and fungal composition analysis is deposited as a project available at https://github.com/jdvaidya/rumenmicrobiotadata. In addition, the sequences are also deposited in ENA under accession number PRJEB22996.

### Statistical Analysis

The significance of potential differences in the relative abundances of bacterial taxa between the different sample groups (e.g., different extraction methods, different rumen fractions) was assessed using the non-parametric rank Mann-Whitney test as implemented in Sci-Phy (Jones et al., 2001). Significance of explanatory variables included in constrained analyses (RDA) was assessed using an unrestricted Monte Carlo permutation test with a total of 999 permutations, and results were visualized in an ordination biplot obtained from Canoco 5. *P*-values were corrected for multiple testing using Bonferroni correction and those lower than 0.05 were regarded as significant.

Two 1-way ANOVA model were fitted separately to DNA yield and quality (260/280 and 260/230) measurements with extraction method (4 levels: RBB, PBB, FDSS and PQIAmini, see Section “DNA Extraction” for detailed explanation of the extraction methods) and rumen fraction (2 levels: fibrous and liquid) as factors, using R software (version 3.0.2). Data was log-transformed before analysis to correct for skewness. The rationale behind the use of two separate one-way ANOVA instead of a two-way ANOVA is that we did not consider the Extraction method × Fraction interaction term, due to large sample heterogeneity (i.e., each of the four cows were fed a different diet).

## Results

### Quality and Quantity of Genomic DNA from Four Extraction Methods

Both RF and FC samples yielded high molecular weight (>3 kb) DNA as confirmed by agarose gel electrophoresis (Supplementary Figure S1). The integrity of the DNA was best for the RBB method, as less DNA degradation was observed compared to the other three methods. Statistical analysis using ANOVA confirmed that the DNA yield was significantly affected by extraction method (*p* < 0.001) and fraction (*p* < 0.001). For the RF samples the highest quantities of DNA were obtained with PBB, which on average yielded 9.0, 3.0 and, 3.5 times more DNA than the RBB, FDSS and PQIAmini methods, respectively (**Table 1**). PBB also yielded the highest quantities of DNA with the FC samples, and yielded 9.5, 2.5 and 2.2 times more DNA than RBB, FDSS and PQIAmini methods, respectively (**Table 1**). Assessment of purity of DNA found that the 260/280 ratio was not affected by extraction (*p* = 0.08) but was affected by fraction (*p* = 0.03). On the other hand, the 260/230 ratio was affected by extraction method (*p* < 0.001) but not affected by fraction (*p* = 0.8). Some DNA extracts of the RF and FC samples had a ratio of absorbance at 260 nm and 280 nm (A_260/280_) that was below 1.8, indicating the presence of contaminants (typically proteins and/or phenol) that absorb at a slightly higher wavelength than DNA (**Table 1**). This was most evident with the PBB method DNA extracts from the FC samples. The A_260/230_ was lower than 2.0 (maximal value for pure DNA) for virtually all of the samples, but in particular for the FDSS DNA extracts. However, all DNA extraction methods provided DNA of sufficient quality and quantity to proceed with PCR based approaches as described in the following sections.

**Table 1:** Purity and yield of genomic DNA extracted from rumen samples taken from cows fed different diets (GS, grass silage and MS, maize silage) and separated into different fractions [rumen fluid (RF) and fibrous content (FC)].

Diet	DNA extraction methods	DNA yield (RF vs. FC)		DNA purity (RF)		DNA purity (FC)	
		RF (μg/ml RF)	FC (μg/g FC)	A260/280	A260/230	A260/280	A260/230
GS100	RBB (I)	6.4	38.7	2.0	2.1	1.7	1.2
	RBB (II)	6.6	28.8	1.9	1.7	1.8	1.3
	PBB (I)	55.9	327.7	1.8	1.6	1.6	1.0
	PBB (II)	50.1	251.5	1.8	1.5	1.6	1.1
	FDSS (I)	20.4	131.4	1.9	0.3	1.9	0.6
	FDSS (II)	23.9	107.4	1.9	0.6	1.9	0.9
	PQIAmini (I)	25.1	83.1	1.8	1.5	1.9	1.8
	PQIAmini (II)	24.4	103.2	1.8	1.7	1.9	1.8
GS67MS33	RBB	6	25.9	2.0	1.8	1.8	1.4
	PBB	57.2	378.2	1.9	1.6	1.7	1.1
	FDSS	21	82.6	1.9	0.5	1.9	0.4
	PQIAmini	20.2	95.2	1.8	1.5	1.9	1.5
GS33MS67	RBB	11.7	30.5	2.1	2.1	1.8	1.2
	PBB	73.7	208.2	1.9	1.5	1.7	1.3
	FDSS	20.9	103.4	1.7	0.2	1.9	0.4
	PQIAmini	27.8	169	1.9	1.7	1.9	1.7
MS100	RBB	7.5	18.1	2.0	1.7	1.9	1.7
	PBB	117.6	187.5	2.0	1.9	1.9	1.9
	FDSS	15.8	96.6	1.9	0.4	1.9	0.5
	PQIAmini	17.7	84.6	1.9	1.5	1.9	1.8

*Four different DNA extraction methods were used (RBB, PBB, FDSS, and PQIAmini), and the DNA extraction of the 100% grass silage diet (GS100) samples was done in duplicate (I & II)*.

### qPCR Analysis of Bacteria, Archaea and Anaerobic Fungi

**FIGURE 1:**
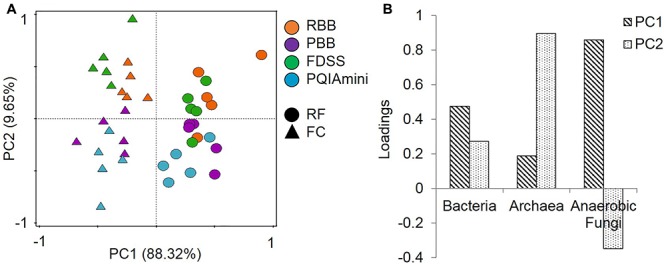
**(A)** Principal component analysis (PCA) of the combined bacterial (16S rRNA gene), archaeal (16S rRNA gene) and anaerobic fungal (5.8S rRNA gene) qPCR data for rumen fluid (RF, Δ) and fibrous content (FC, aaa) samples. The GS100 diet has duplicate DNA extracts presented as individual datapoints. The percentages provided at the axes indicate the variation explained. **(B)** The corresponding loadings for the principal components indicate that anaerobic fungi are the major cause of sample separation in PC1, and archaea in PC2.

All DNA extracts from RF and FC samples were used for qPCR analysis of total bacteria, archaea and anaerobic fungi (Supplementary Figure S2). The PCA of the qPCR data revealed separate clustering of the FC and RF fractions in PC1 (**Figure 1A**). These two clusters were separated by anaerobic fungal 5.8S rRNA gene concentrations along the first principal component axis (PC1). There was also evidence of clustering of the extraction methods in the second principal component axis (PC2), with the RBB and FDSS methods clustered to the top half of the plot and PBB and PQIAmini to the bottom (**Figure 1A**). Archaeal 16S rRNA gene concentrations were associated with the separation of these two clusters in PC2 (**Figure 1B**).

### Impact of DNA Extraction Methods and Fractions on Observed Bacterial Community Composition

On average only 26.3% of the annotations for the bacterial taxa included genus level identification. Therefore, mainly the OTU and family level (average of 56.2% annotation) was used in the data analysis. Weighted UniFrac distance based PCoA at the OTU-level showed that the bacterial communities observed in RBB, FDSS, and PQIAmini-derived extracts generally grouped together, whereas the bacterial communities associated with PBB-derived extracts clustered separately (Supplementary Figure S3A). This was not seen in the unweighted UniFrac distance based PCoA, however, samples appeared to cluster more by rumen fraction instead (Supplementary Figures S3A,B).

**FIGURE 2:**
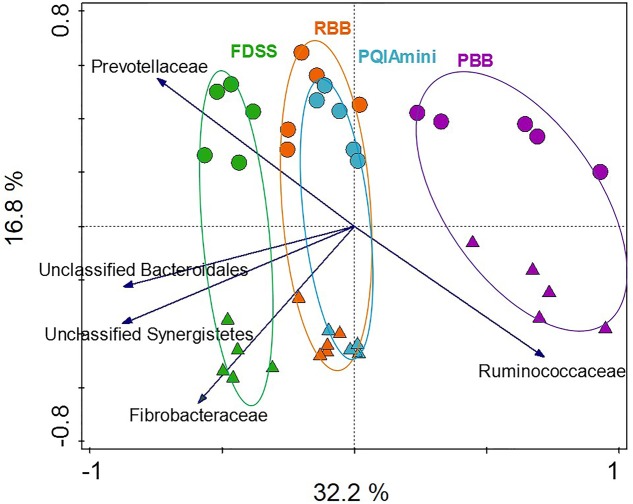
Redundancy analysis triplot (RDA) showing the relationship between the top five family level phylogenetic groupings, the variation of which is most strongly associated with DNA extraction methods and fractions. The canonical axes are labeled with percentage of total variance explained (%). Arrow length indicates the variance explained by extraction methods and fractions. The GS100 diet has duplicate DNA extracts presented as individual datapoints.

In order to test to what extent different extraction methods and rumen fractions contributed to explaining the observed variation in bacterial community composition, redundancy analysis (RDA) was applied using family level relative abundance data. This analysis showed that the PBB (*p* = 0.012) and FDSS (*p* = 0.024) DNA extraction methods were separated relative to RBB and PQIAmini on the first canonical axis (**Figure 2**). On the second canonical axis samples were separated by fraction (*p* = 0.012) (**Figure 2**). Ruminococcaceae appeared to be positively associated with the PBB method and the FC. The following three families were positively associated with the FDSS method and FC: Fibrobacteraceae, Unclassified Synergistetes and Unclassified Bacteroidales (**Figure 2**). The Prevotellaceae were positively associated with the FDSS method and RF fraction.

#### Bacterial Community Analysis

**FIGURE 3:**
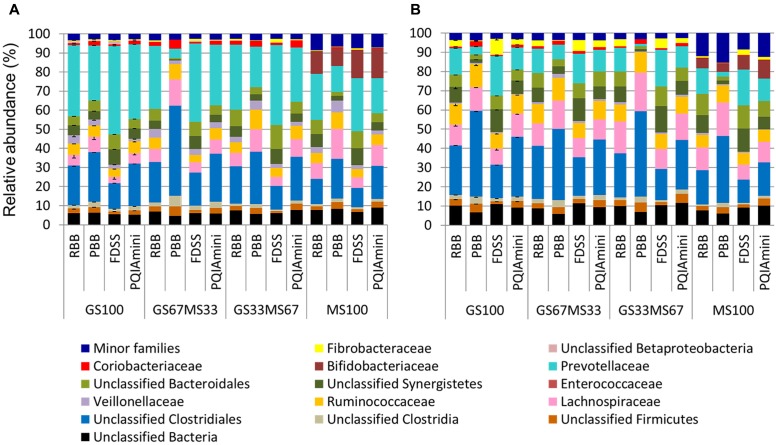
Bacterial family level composition of different DNA extracts obtained from rumen fluid **(A)** and fibrous content **(B)** samples from dairy cows each fed different ratios of grass silage (GS) to maize silage (MS), e.g., GS67MS33 is a diet containing 67% grass silage and 33% maize silage. All the stacked bars represent individual sample data except for GS100 which represents the mean of two different DNA extracts (error bars represent the standard deviation). All family level phylogenetic groupings > 1% are shown individually, with those <1% summed together and presented as minor families.

**FIGURE 4:**
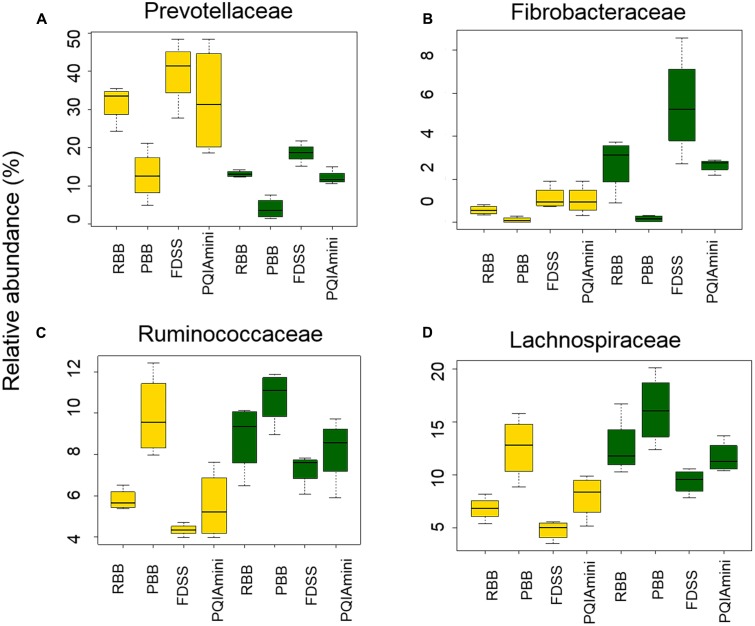
The effect of DNA extraction method (RBB, PDD, FDSS, and PQIAmini) on the relative abundance of the bacterial families Prevotellaceae **(A)**, Fibrobacteraceae **(B)**, Ruminococcaceae **(C)**, and Lachnospiraceae **(D)** in rumen fluid (yellow) and fibrous content (green) samples. The boxplots represent the data from 5 observations per rumen fraction, and show the 25th, 50th and 75th percentiles, with whiskers showing the extremes of the data.

A more detailed compositional analysis of RF and FC samples showed that the rumen bacterial community consisted of 15 phyla (data not shown), among which on average Firmicutes (46.9 ± 16.1% RF, 39.7 ± 15.7% FC) and Bacteroidetes (58.2 ± 15.7% RF, 26.9 ± 11.2% FC) were most predominant. The bacterial profiles of RF and FC fractions appeared to be very distinct at the family level (**Figures 3A,B**). Overall, the relative abundance of Prevotellaceae was significantly higher in RF samples than in FC samples (*p* = 0.001; **Figure 4A** and Supplementary Table S1A) but was not significantly affected by any of the DNA extraction methods (*p* > 0.05; **Figure 4A** and Supplementary Table S1B). The relative abundance of Fibrobacteraceae was higher in FC compared to RF (*p* = 0.020; **Figure 4B** and Supplementary Table S1A), and was found to be higher (*p* = 0.028) in extracts obtained using the FDSS method as compared to the PBB method (**Figure 4B** and Supplementary Table S1B). The RBB method also resulted in DNA extracts with a higher relative abundance of Fibrobacteraceae in comparison to the PBB method (*p* = 0.038; **Figure 4B** and Supplementary Table S1B). Differences were also observed between RF and FC fractions for Ruminococcaceae. FC samples had significantly higher relative abundances of Ruminococcaceae compared to RF samples (*p* = 0.040; **Figure 4C** and Supplementary Table S1A). The PBB extraction method gave significantly higher relative abundances of Ruminococcaceae compared to the FDSS method (*p* = 0.038; **Figure 4C** and Supplementary Table S1B). Members of the Lachnospiraceae appeared to be predominant in both RF and FC samples, and their relative abundance in FC samples was significantly higher than those in RF samples (*p* = 0.006; **Figure 4D** and Supplementary Table S1A). However, there was no effect of DNA extraction methods on Lachnospiraceae (**Figure 4D** and Supplementary Table S1B). Finally, relative abundances of two other minor (<1%) families (Anaerolinaceae and Halomonadaceae) were significantly affected by DNA extraction methods (Supplementary Table S1B) and one minor family (Desulfobulbaceae) was affected by fraction (Supplementary Table S1A).

At the genus level, *Selenomonas, Succiniclasticum, Ruminococcus, Prevotella, Butyrivibrio, Paraeggerthella, Fibrobacter, Desulfobulbus, Pseudobutyrivibrio, Syntrophococcus*, and *Oscillibacter* significantly differed in relative abundance when comparing RF and FC (*p* < 0.05; Supplementary Table S1A). The genera with higher relative abundance in RF compared to FC fraction were *Desulfobulbus, Succiniclasticum, Paraeggerthella, Prevotella*, and *Selenomonas*, whereas *Syntrophococcus, Pseudobutyrivibrio, Butyrivibrio, Oscillibacter, Ruminococcus* and *Fibrobacter* were significantly higher in their relative abundance in the FC fraction as compared to the RF fraction. In contrast, only the genus *Fibrobacter* was found to be significantly affected by DNA extraction method. The relative abundance of *Fibrobacter* was higher in FDSS extracts compared to those prepared using the PBB method (*p* = 0.038), and higher also in the RBB extracts compared to the PBB (*p* = 0.038) (Supplementary Table S1B).

#### Bacterial Diversity and Richness

**FIGURE 5:**
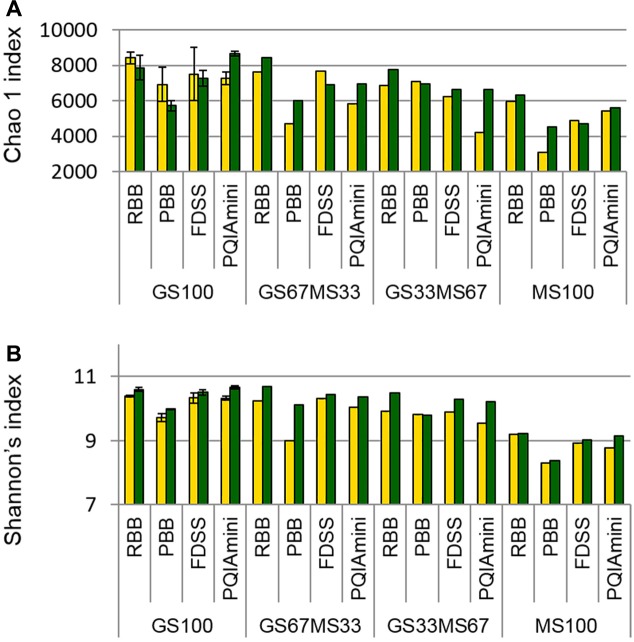
Chao1 richness index **(A)** and Shannon’s diversity index **(B)** values for all four DNA extraction methods (RBB, PBB, FDSS, and PQIAmini) applied to rumen fluid (yellow) and fibrous content (green) samples from four dairy cows each fed different ratios of grass silage (GS) to maize silage (MS), e.g., GS67MS33 is a diet containing 67% grass silage and 33% maize silage. The GS100 samples represent the mean of two different DNA extracts, and the error bars represent their standard deviation.

Estimates of bacterial sequence richness and diversity were calculated at the OTU level to assess if these parameters were affected by fraction or DNA extraction method. The PBB extracts from RF and FC fractions of GS67MS33 and MS100 fed cows appeared to generally have the lowest bacterial richness (total number of OTUs present in a community) as calculated by the Chao1 index than the corresponding RBB, FDSS and PQIAmini RF and FC extracts (**Figure 5A**). A similar trend of the PBB extracts was also seen for diversity (Shannon’s index, **Figure 5B**). Within the GS100 sample, the Chao1 richness index generally showed higher variability within RF than in FC samples (**Figure 5A**). On the other hand, the technical replicates for GS100 appeared to give similar values throughout Shannon’s index analyses (**Figure 5B**). The RF samples seemed to have lower Shannon’s index values compared to the FC samples, which was not always the case with Chao1 index values.

#### Archaeal Community Analysis

**FIGURE 6:**
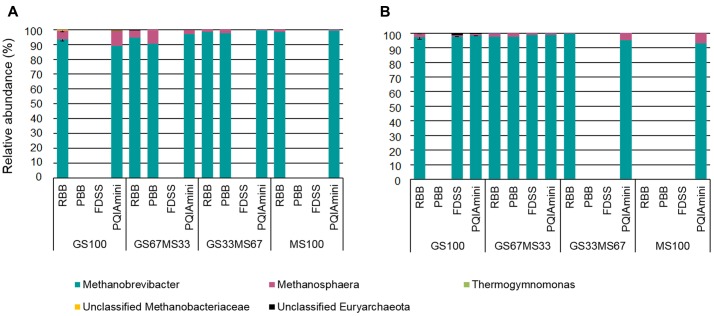
Relative abundance of archaeal taxa at genus level within rumen fluid **(A)** and fibrous content **(B)** samples from dairy cows each fed different ratios of grass silage (GS) to maize silage (MS), e.g., GS67MS33 is a diet containing 67% grass silage and 33% maize silage. All samples were subjected to each of the four different DNA extraction methods (RBB, PBB, FDSS, and PQIAmini). Missing bars indicate that it was not possible to generate an archaeal PCR product for sequencing. Error bars for the GS100 samples represent the standard deviation associated with two different extracts, except for the PQIAmini extracted GS100 rumen fluid sample **(A)** where *n* = 1.

The RF and FC samples were analyzed to identify the rumen archaea associated with the different fractions, and how the different DNA extraction methods influenced their detection (**Figures 6A,B**). Some of the DNA extracts did not yield amplicons for sequencing, despite the fact that all samples were successfully amplified in the archaeal 16S rRNA qPCR. Furthermore, the PCR failure could also not be directly correlated with either the fraction, sample source (cow/diet) or any of the DNA extraction methods. The FDSS method, however, consistently failed with all the RF samples (**Figure 6A**).

Two families belonging to the phylum Euryarchaeota, i.e., Methanobacteriaceae and Thermoplasmata-incertae-sedis represented the majority of the sequences. Within the Methanobacteriaceae, two known genera were detected, *Methanobrevibacter* (∼83% to 98%) and *Methanosphaera* (∼1% to 4%). An unidentified genus within the Methanobacteriaceae was also detected (<1%) (**Figures 6A,B**). Within Thermoplasmatales-incertae-sedis, only the genus *Thermogymnomonas* (<1%) was identified. Of the samples for which sequence information could be generated, there was no consistent difference in the relative abundances of archaeal genera found relative to the different DNA extraction methods. However, from the two fractions there was generally more *Methanosphaera* seen in the RF as compared to the FC.

#### Fungal Community Analysis

Pyrosequencing analysis of amplified fungal ITS regions revealed the presence of several fungal phyla in both the RF and FC samples, and included aerobic (Ascomycota and Basidiomycota) as well as anaerobic fungi (Neocallimastigomycota). Unidentified fungal taxa (including but not differentiating between unidentified anaerobic and aerobic) and not assigned fungi were dominant (**Tables 2A,B**). The identified anaerobic fungi represented less than 1% of the reads in the RF samples (**Table 2A**). The FC samples on the other hand were characterized by much higher relative abundances of the anaerobic fungi, which were represented by four genera: *Cyllamyces* (0–3.2%), *Anaeromyces* (0–5.2 %), *Neocallimastix* (0–8.1%) and *Piromyces* (0–31.5%) (**Table 2B**). Due to the limited and variable number of anaerobic fungal reads, an in depth analysis of this phylum relative to the DNA extraction method was not possible.

**Table 2:** Anaerobic fungal composition at genus level for the the rumen fluid **(A)** and fibrous content **(B)** samples from dairy cows each fed different ratios of grass silage (GS) to maize silage (MS), e.g., GS67MS33 is a diet containing 67 % grass silage and 33 % maize silage.

(A)					
**Diet**	**DNA extraction methods**	**Relative abundance (%)**					
		**Piromyces**	**Neocallimastix**	**Cyllamyces**	**Anaeromyces**	**Orpinomyces**	**Unidentified fungi**

GS100	RBB I	0.05	nd	0.1	nd	nd	71.41
	RBB II	nd^∗^	nd	0.21	nd	nd	52.22
	PBB I	nd	nd	0.07	nd	nd	80.24
	PBB II	nd	nd	0.19	nd	nd	78.09
	FDSS I	0.28	0.09	0.28	nd	nd	63.88
	FDSS II	nd	0.08	nd	nd	nd	79.97
	PQIAmini I	nd	nd	nd	nd	nd	86.47
	PQIAmini II	nd	nd	nd	nd	nd	88.59
GS67MS33	RBB	nd	nd	nd	nd	nd	55.11
	PBB	nd	nd	nd	nd	nd	31.84
	FDSS	nd	nd	nd	0.03	nd	76.36
	PQIAmini	nd	nd	nd	nd	nd	66.77
GS33MS67	RBB	0.16	0.31	nd	nd	nd	31.42
	PBB	nd	nd	nd	nd	nd	60.51
	FDSS	0.1	nd	0.05	0.05	nd	35.23
	PQIAmini	nd	nd	nd	nd	nd	53.51
MS100	RBB	nd	0.03	nd	nd	nd	66.4
	PBB	nd	0.04	nd	nd	nd	32.77
	FDSS	nd	0.02	nd	nd	nd	75.03
	PQIAmini	nd	nd	nd	nd	nd	75.18

**(B)**					

GS100	RBB I	nd^∗^	0.99	nd	nd	nd	2.97
	RBB II	nd	nd	nd	nd	nd	19.2
	PBB I	0.06	nd	nd	nd	nd	16.56
	PBB II	2.81	1.25	2.5	nd	nd	14.38
	FDSS I	1.59	0.79	3.17	nd	nd	6.35
	FDSS II	1.23	3.7	1.23	nd	nd	13.58
	PQIAmini I	1.54	3.08	3.08	nd	nd	27.69
	PQIAmini II	1.16	2.33	1.16	1.16	nd	30.23
GS67MS33	RBB	0.86	3.45	0.86	5.17	nd	18.1
	PBB	1.56	nd	nd	1.56	nd	73.44
	FDSS	1.22	nd	2.44	0.61	nd	6.1
	PQIAmini	nd	0.88	0.88	nd	nd	36.84
GS33MS67	RBB	nd	nd	nd	nd	0.6	1.79
	PBB	0.73	1.22	nd	nd	nd	1.46
	FDSS	1.61	8.06	nd	nd	nd	nd
	PQIAmini	0.83	1.65	nd	nd	nd	7.44
MS100	RBB	nd	0.37	nd	nd	nd	42.61
	PBB	31.51	1.37	nd	nd	nd	39.73
	FDSS	nd	nd	nd	nd	nd	17.89
	PQIAmini	nd	0.54	nd	nd	nd	63.39

*The unidentified fungi represent aerobic and anaerobic fungi that could not be classified to the genus level, and the remaining fraction (not indicated in the table) belongs to fungi that could not be classified at the phylum level. DNA extraction technical replicates for the GS100 samples are denoted I and II. ^∗^nd, not detected*.

## Discussion

### DNA Quantity, Purity and Integrity

The different cell wall composition and structure of bacteria, archaea and fungi largely determines their susceptibility to mechanical or enzymatic lysis methods (Fredricks et al., 2005; Henderson et al., 2013). In this study, all the methods employed mechanical disruption of cells by bead beating, albeit with differences in agitation times and type of beads. Several studies have shown that disruption of bacteria with tough cell walls, such as those belonging to the phyla Firmicutes and Actinobacteria, is more efficient with a mechanical approach than by an enzyme-based protocol (Lazarevic et al., 2013). In the present study, all four DNA extraction methods yielded high molecular weight DNA (>3 kb), from both RF and FC fractions based on agarose gel analysis but the mechanical disruption caused shearing of DNA to different extents. The RBB method yielded the most intact genomic DNA compared to the PBB, FDSS and PQIAmini methods. Although the RBB method employs two rounds of bead beating in the presence of high concentrations of SDS, salt and EDTA, the physical damage of DNA is minimized by removing the lysate from the first round of bead beating to a new micro centrifuge tube followed by a second bead beating step to lyse any remaining intact cells. The DNA yields for RBB were lower compared to the other methods assessed. DNA yields previously reported for fecal samples (10–30 μg/g feces: Zoetendal et al., 2006) were slightly higher compared to the range observed for RF, and lower than that observed for the FC.

It has previously been shown that different agitation speeds can affect DNA extraction, as samples subjected to disruption at 4,800 rpm yielded more DNA than those subjected to 2,400 rpm (Fujimoto et al., 2004). In our study, although RBB and PQIAmini methods both used an agitation speed of 5.5 m/s, the DNA yields for PQIAmini were 3.0 and 3.8 times higher as compared to the RBB method for RF and FC samples, respectively. In this case, either the reagents used during lysis or the different disruption times (3 × 1 min for RBB and 3 × 45 s for PQIAmini) might be responsible for the different DNA yields obtained from these two methods for RF and FC.

In this study, the average 260/230 ratios observed for RF (1.4 ± 0.6) and FC (1.2 ± 0.5) samples indicated the presence of humic acids or guanidine carried over during the washing steps of the silica columns and the beads. For some extraction methods, the 260/230 ratio seemed particularly low for FC samples as compared to RF samples, presumably due to impurities associated with the lignocellulose components of a plant fibrous material rich in aromatic ring structures similar to humic acids. The 260/230 ratio for samples extracted with the FDSS method were the lowest as compared to the other extraction methods (**Table 1**). The FDSS protocol has previously been reported to give high DNA yields with soil samples, but still containing contaminants such as humic acid residues (Devi et al., 2015). It is also important to note that phenol-based DNA extraction methods, including the PBB and PQIAmini methods, can give higher 260/280 ratios as any residual phenol absorbs at 280nm. Nevertheless, no PCR inhibition was evident in any of the qPCR analyses performed. For PCR based community analyses, we observed that bacterial and fungal pyrosequencing PCR was successful. However, the pyrosequencing PCR targeting archaeal 16S rRNA genes did not work for all samples. This is presumably due to the lower number of PCR cycles used with this method (25 cycles) compared to that of the bacteria (30 cycles) and fungi (35 cycles). Noteworthy is the observation that only PQIAmini DNA extracts generated archaeal amplicons for both fractions for all the samples.

### Pyrosequencing Analysis

#### Bacteria Community Analysis

The clustering of bacterial communities was distinct for the PBB method and FDSS method as compared to the RBB and PQIAmini methods (**Figure 2**). From the PCoA plots (Supplementary Figure S3), the latter three methods however, had a gradual shift of the bacterial communities between methods, suggesting that all four DNA extraction methods had an effect on the observed bacterial community structure to some extent. Further analysis of the data confirmed that DNA extraction method affected the relative abundances of various families and genera.

The predominant phyla detected in this study were Firmicutes and Bacteroidetes, which is in line with other bovine rumen based studies (De Menezes et al., 2011; Li et al., 2014; Huws et al., 2016). The predominant family level taxa belonging to Firmicutes in the RF fraction were: Ruminococcaceae, Lachnospiraceae and unclassified Clostridiales. Within the Bacteroidetes, Prevotellaceae was the predominant family. These observed families were in line with a previous study (Mao et al., 2015). The FC fraction showed a significantly higher relative abundance of Fibrobacteraceae, Ruminococcaceae, and Lachnospiraceae compared to the RF fraction. These three families were also pre-dominant in our study, which is in accordance with another bovine rumen microbiota study (McCann et al., 2014). Ruminococcaceae were observed at significantly higher relative abundances in extracts prepared with the PBB method as compared to the FDSS method. This suggests that the PBB method was more effective in lysing these cells, or conversely that it was less effective in lysing cells of other microbial groups since the data is based on relative abundance. Lachnospiraceae, on the other hand, was not affected by any extraction method. In a study from Fouts et al. (2012), two members of the Lachnospiraceae, namely *Butyrivibrio* and *Blautia*, were reported to have significantly higher relative abundance in the FC as compared to the RF fraction. Partly in agreement with this, we observed a fraction effect for *Butyrivibrio* but not for *Blautia*. There was a significant decrease of the family Fibrobacteraceae for the PBB method as compared to the RBB and FDSS for the FC fraction samples, indicating that the PBB method was less effective in extracting Fibrobacter DNA compared to other methods. Similarly, for many other genera like *Selenomonas, Succiniclasticum, Ruminococcus, Prevotella, Paraeggerthella, Syntrophococcus, Fibrobacter, Oscillibacter, Desulfobulbus* and *Pseudobutyrivibrio* we observed a fraction effect indicating a distinct separation of microbial communities associated with RF and FC fractions, which is in line with the bovine rumen study of Fouts et al. (2012). This fraction effect might be explained by the different feed components available in the RF and FC fractions (insoluble polymers versus soluble monomers), as well as the difference in ability of cells to adhere to the plant fibers.

Together, these data reinforce the notion that not all bacterial community members and rumen fractions are equally affected by the tested extraction methods, making it difficult to come up with informed decisions as to which extraction method generates DNA that is most representative of the rumen bacterial community. To this end, synthetic communities of defined bacterial composition could provide additional insight, in analogy to defined mock communities assembled at the DNA level that have been used to assess the influence of different steps during molecular community assessment (Ramiro-Garcia et al., 2016). One could argue, however, that such synthetic communities would not sufficiently represent *in vivo* rumen conditions, especially for the FC fraction, and thus, particular attention will need to be paid to the design of such analyses.

#### Archaea Community Analysis

The DNA extracts obtained from the four extraction methods amplified well for qPCR but when used for 16S rRNA gene-based archaeal community assessment, not all the samples yielded PCR products. One of the possible reasons for this observation, as mentioned above, is the lower number of PCR cycles used for this particular taxon. Furthermore, archaeal diversity was found to be very limited compared to bacteria. A previous study on the comparison of DNA extraction methods on rumen fractions revealed *Methanobrevibacter* spp. as the most dominant methanogen from all extraction methods applied (Henderson et al., 2013). In the Henderson et al. (2013) study one universal primer pair was used to simultaneously amplify the 16S and 18S rRNA genes of bacteria, archaea and ciliate protozoa. This type of approach would avoid the issues encountered in this study with limited amplification of the archaeal 16S rRNA gene in some samples. A universal 16S rRNA sequencing approach, simultaneously amplifying 16S rRNA genes of both the bacteria and archaea, could also be used (Van Lingen et al., 2017). A potential drawback of a universal primer approach could be that if bacteria are more abundant, archaea might not be detected at all. As a consequence, attention should be paid to an appropriate sequencing depth that would safeguard detection and identification of archaeal populations of relative abundances > 1%.

The relative distribution of different archaeal populations has previously been shown to be affected by several factors such as diet, host age or species, season and geographical region (Huang et al., 2016). In this study, in the samples for which a PCR product could be generated, the genera *Methanobrevibacter* followed by *Methanosphaera* were the dominant archaeal taxa in all the RF and FC samples. Similar to our results, both *Methanobrevibacter* and *Methanosphaera* were found to be conserved members of the methanogenic population in other bovine studies which focused on physiological interactions within the rumen microbial food web (Janssen and Kirs, 2008; Henderson et al., 2013; De Mulder et al., 2016). *Methanobrevibacter* species can utilize H_2_, CO_2_ and formate, whereas *Methanosphaera* species can produce CH_4_ only via reduction of methanol with H_2_ (Carberry et al., 2014). From a recent study by Van Lingen et al. (2016) it was shown that there is no benefit for the methane producers if H_2_ or formate are consumed, as there is no energetic limitation due to H_2_/formate accumulation in the rumen.

Interestingly in the current study, qPCR showed high numbers of methanogenic archaea in the FC fraction as compared to the RF fraction. This is consistent with the results obtained in a recent study by De Mulder et al. (2016) indicating that the methanogenic archaea make up an intrinsic part of the solid fraction in the cow rumen. The presence of archaea in the FC fraction, however, is not surprising considering the close physical and metabolic interactions of methanogens with anaerobic fungi, which extensively colonize and invade rumen FC (Cheng et al., 2009; Jin et al., 2011). It was noted in this study that *Methanosphaera* seemed to have a lower relative abundance in the FC fraction compared to the RF fraction, however, further work is needed to verify this due to the limited number of biological samples used in this study.

#### Fungal Community Analysis

Sequences from anaerobic fungi (Neocallimastigomycota phylum) were obtained from five genera: *Piromyces, Anaeromyces, Neocallimastix, Cyllamyces* and *Orpinomyces*. These fungi are involved in the degradation of the lignocellulose fraction of plant material in the rumen (Kittelmann et al., 2012). In line with this, a larger proportion of anaerobic fungal reads was on average observed in the FC fraction as compared to the RF fraction in the fungal community analysis. This is also consistent with the qPCR analysis, which revealed a higher abundance of anaerobic fungi in FC relative to RF fractions. In our study, the anaerobic fungal community in FC fraction samples was mainly composed of the genera *Cyllamyces* (2 to 3%), *Neocallimastix* (1 to 3%) and *Piromyces* (1 to 2%) with sequences assigned to *Orpinomyces* only detected in the GS33MS67 FC sample subjected to the RBB method (**Table 2B**). On the other hand in RF samples, no sequences from *Orpinomyces* were detected and a more limited amount of all of the other genera were detected compared to FC samples (**Table 2A**). The overall higher detection of anaerobic fungal genera in FC, as compared to the RF fraction, is likely to be due to the motile zoospores being only transiently present within RF for a short time after feeding (Orpin, 1974, 1975, 1976, 1977; Griffith et al., 2009).

Besides the identification of the five genus level groups mentioned above belonging to Neocallimastigomycota, we observed a large number of unidentified fungi belonging to both anaerobic and aerobic fungi (**Tables 2A,B**) as well as a high proportion of sequences that could not be further assigned to any phylum. As Neocallimastigomycota are considered to be the key fungal phylum relative to rumen function, community assessment of this specific community using targeted anaerobic fungal primers would provide a better approach, as amplification of aerobic fungi associated with ingested feed and water would be avoided. The larger read depth this would generate would also improve the ability to interpret the impact of different experimental factors on the taxa within the phylum, particularly as there is an increasing evidence of anaerobic fungal niche differentiation within the rumen (Griffith et al., 2009). Furthermore, a custom ITS1 database is also available specifically for the Neocallimastigomycota phylum (Koetschan et al., 2014).

## Conclusion

DNA extraction methods clearly have an impact on the outcome of downstream rumen microbial community analyses, including relative abundances of specific community members. From this study, this effect was evident with the bacterial community, however, no single extraction method could be concluded as being ineffective. Rather, every extraction method presented its own strengths and weaknesses in observing specific bacterial families. DNA extracted using the PBB method resulted in higher relative abundance of Ruminococcaceae than the FDSS method, whereas relative abundance of Fibrobacteraceae was lower compared to the RBB method. Whilst the effect of DNA extraction method was limited compared to that of rumen fraction, differences due to both DNA extraction method and fraction were observed for certain taxa. Further investigation is needed to determine if this is due to an issue with the physical nature of the different fractions, or merely due to the inherent differences in the microbes present within the fractions. Furthermore, careful selection of the microbial community assessment approach is needed to avoid the issues encountered within this study with respect to archaea and anaerobic fungi. Archaeal 16S rRNA gene barcoded amplicons are best generated in combination with other taxa (bacteria or bacteria and protozoa), whilst anaerobic fungi should be generated with phylum specific primers rather than those designed to cover the entire fungal kingdom. In summary, the comprehensive assessment of observed communities of bacteria, archaea and fungi described here provides insight into a rational basis for selecting an optimal methodology to obtain a representative picture of the rumen microbiome.

## Author Contributions

JV, BvdB, CP, and HS designed the experiment. JV, BvdB, and SvG performed the sampling. JV, BvdB, and JB performed the experiments. JV, BvdB, JE, JB, SvG, ES, CP, and HS performed analysis and interpretation of data. BvdB, JE, CP, and HS supervised the work. JV and JE wrote the manuscript. All authors read, improved and approved the final manuscript.

## Conflict of Interest Statement

The authors declare that the research was conducted in the absence of any commercial or financial relationships that could be construed as a potential conflict of interest. The handling Editor declared a past co-authorship with one of the authors JE.

## References

[B1] Abarenkov K. Henrik Nilsson R. Larsson K. H. Alexander I. J. Eberhardt U. Erland S. (2010). The UNITE database for molecular identification of fungi–recent updates and future perspectives. *New Phytol.* 186 281–285. 10.1111/j.1469-8137.2009.03160.x 20409185

[B2] Altschul S. F. Gish W. Miller W. Myers E. W. Lipman D. J. (1990). Basic local alignment search tool. *J. Mol. Biol.* 215 403–410. 10.1016/S0022-2836(05)80360-2 2231712

[B3] Bauchop T. (1979). Rumen anaerobic fungi of cattle and sheep. *Appl. Environ. Microbiol.* 38 148–158. 16345408 10.1128/aem.38.1.148-158.1979 PMC243449

[B4] Caporaso J. G. Kuczynski J. Stombaugh J. Bittinger K. Bushman F. D. Costello E. K. (2010). QIIME allows analysis of high-throughput community sequencing data. *Nat. Methods* 7 335–336. 10.1038/nmeth.f.303 20383131 PMC3156573

[B5] Carberry C. A. Waters S. M. Kenny D. A. Creevey C. J. (2014). Rumen methanogenic genotypes differ in abundance according to host residual feed intake phenotype and diet type. *Appl. Environ. Microbiol.* 80 586–594. 10.1128/AEM.03131-13 24212580 PMC3911112

[B6] Cheng Y. F. Edwards J. E. Allison G. G. Zhu W. Y. Theodorou M. K. (2009). Diversity and activity of enriched ruminal cultures of anaerobic fungi and methanogens grown together on lignocellulose in consecutive batch culture. *Bioresour. Technol.* 100 4821–4828. 10.1016/j.biortech.2009.04.031 19467591

[B7] Creevey C. J. Kelly W. J. Henderson G. Leahy S. C. (2014). Determining the culturability of the rumen bacterial microbiome. *Microb. Biotechnol.* 7 467–479. 10.1111/1751-7915.12141 24986151 PMC4229327

[B8] Daims H. Bruhl A. Amann R. Schleifer K. H. Wagner M. (1999). The domain-specific probe EUB338 is insufficient for the detection of all Bacteria: development and evaluation of a more comprehensive probe set. *Syst. Appl. Microbiol.* 22 434–444. 10.1016/S0723-2020(99)80053-8 10553296

[B9] De Menezes A. B. Lewis E. O’Donovan M. O’Neill B. F. Clipson N. Doyle E. M. (2011). Microbiome analysis of dairy cows fed pasture or total mixed ration diets. *FEMS Microbiol. Ecol.* 78 256–265. 10.1111/j.1574-6941.2011.01151.x 21671962

[B10] De Mulder T. Goossens K. Peiren N. Vandaele L. Haegeman A. De Tender C. (2016). Exploring the methanogen and bacterial communities of rumen environments: solid adherent, fluid and epimural. *FEMS Microbiol. Ecol.* 93:fiw251. 10.1093/femsec/fiw251 28011597

[B11] Dehority B. A. (1991). Effects of microbial synergism on fibre digestion in the rumen. *Proc. Nutr. Soc.* 50 149–159. 10.1079/PNS19910026 1661009

[B12] Devi S. G. Fathima A. A. Radha S. Arunraj R. Curtis W. R. Ramya M. (2015). A rapid and economical method for efficient DNA extraction from diverse soils suitable for metagenomic applications. *PLOS ONE* 10:e0132441. 10.1371/journal.pone.0132441 26167854 PMC4500551

[B13] Dojka M. A. Hugenholtz P. Haack S. K. Pace N. R. (1998). Microbial diversity in a hydrocarbon- and chlorinated-solvent-contaminated aquifer undergoing intrinsic bioremediation. *Appl. Environ. Microbiol.* 64 3869–3877. 9758812 10.1128/aem.64.10.3869-3877.1998 PMC106571

[B14] Edwards J. E. Kingston-Smith A. H. Jimenez H. R. Huws S. A. Skøt K. P. Griffith G. W. (2008). Dynamics of initial colonization of nonconserved perennial ryegrass by anaerobic fungi in the bovine rumen. *FEMS Microbiol. Ecol.* 66 537–545. 10.1111/j.1574-6941.2008.00563.x 18673390

[B15] Edwards J. E. McEwan N. R. Travis A. J. Wallace R. J. (2004). 16S rDNA library-based analysis of ruminal bacterial diversity. *Antonie Van Leeuwenhoek* 86 263–281. 10.1023/B:ANTO.0000047942.69033.24 15539930

[B16] Fliegerova K. Tapio I. Bonin A. Mrazek J. Callegari M. L. Bani P. (2014). Effect of DNA extraction and sample preservation method on rumen bacterial population. *Anaerobe* 29 80–84. 10.1016/j.anaerobe.2013.09.015 24125910

[B17] Fouts D. E. Szpakowski S. Purushe J. Torralba M. Waterman R. C. MacNeil M. D. (2012). Next generation sequencing to define prokaryotic and fungal diversity in the bovine rumen. *PLOS ONE* 7:e48289. 10.1371/journal.pone.0048289 23144861 PMC3492333

[B18] Fredricks D. N. Smith C. Meier A. (2005). Comparison of six DNA extraction methods for recovery of fungal DNA as assessed by quantitative PCR. *J. Clin. Microbiol.* 43 5122–5128. 10.1128/JCM.43.10.5122-5128.2005 16207973 PMC1248488

[B19] Fujimoto S. Nakagami Y. Kojima F. (2004). Optimal bacterial DNA isolation method using bead-beating technique. *Mem. Kyushu Univ. Dep. Health Sci. Med. Sch.* 3 33–38. 22666455

[B20] Gantner S. Andersson A. F. Alonso-Saez L. Bertilsson S. (2011). Novel primers for 16S rRNA-based archaeal community analyses in environmental samples. *J. Microbiol. Methods* 84 12–18. 10.1016/j.mimet.2010.10.001 20940022

[B21] Griffith G. W. Ozkose E. Theodorou M. K. Davies D. R. (2009). Diversity of anaerobic fungal populations in cattle revealed by selective enrichment culture using different carbon sources. *Fungal Ecol.* 2 87–97. 10.1016/j.funeco.2009.01.005

[B22] Gruninger R. J. Puniya A. K. Callaghan T. M. Edwards J. E. Youssef N. Dagar S. S. (2014). Anaerobic fungi (phylum Neocallimastigomycota): advances in understanding their taxonomy, life cycle, ecology, role and biotechnological potential. *FEMS Microbiol. Ecol.* 90 1–17. 10.1111/1574-6941.12383 25046344

[B23] Guss A. M. Roeselers G. Newton I. L. Young C. R. Klepac-Ceraj V. Lory S. (2011). Phylogenetic and metabolic diversity of bacteria associated with cystic fibrosis. *ISME J.* 5 20–29. 10.1038/ismej.2010.88 20631810 PMC3105664

[B24] Haas B. J. Gevers D. Earl A. M. Feldgarden M. Ward D. V. Giannoukos G. (2011). Chimeric 16S rRNA sequence formation and detection in Sanger and 454-pyrosequenced PCR amplicons. *Genome Res.* 21 494–504. 10.1101/gr.112730.110 21212162 PMC3044863

[B25] Haitjema C. H. Solomon K. V. Henske J. K. Theodorou M. K. O’Malley M. A. (2014). Anaerobic gut fungi: advances in isolation, culture, and cellulolytic enzyme discovery for biofuel production. *Biotechnol. Bioeng.* 111 1471–1482. 10.1002/bit.25264 24788404

[B26] Hamady M. Walker J. J. Harris J. K. Gold N. J. Knight R. (2008). Error-correcting barcoded primers for pyrosequencing hundreds of samples in multiplex. *Nat. Methods* 5 235–237. 10.1038/nmeth.1184 18264105 PMC3439997

[B27] Henderson G. Cox F. Kittelmann S. Miri V. H. Zethof M. Noel S. J. (2013). Effect of DNA extraction methods and sampling techniques on the apparent structure of cow and sheep rumen microbial communities. *PLOS ONE* 8:e74787. 10.1371/journal.pone.0074787 24040342 PMC3770609

[B28] Huang X. D. Martinez-Fernandez G. Padmanabha J. Long R. Denman S. E. McSweeney C. S. (2016). Methanogen diversity in indigenous and introduced ruminant species on the Tibetan plateau. *Archaea* 2016:5916067. 10.1155/2016/5916067 27274707 PMC4864563

[B29] Hungate R. E. (ed.) (1966a). “Chapter II - The rumen bacteria,” in *The Rumen and its Microbes* (Cambridge, MA: Academic Press), 8–90. 10.1016/B978-1-4832-3308-6.50005-X

[B30] Hungate R. E. (ed.) (1966b). “Chapter III - The rumen protozoa,” in *The Rumen and its Microbes* (Cambridge, MA: Academic Press), 91–147. 10.1016/B978-1-4832-3308-6.50006-1

[B31] Huws S. A. Edwards J. E. Creevey C. J. Rees Stevens P. Lin W. Girdwood S. E. (2016). Temporal dynamics of the metabolically active rumen bacteria colonizing fresh perennial ryegrass. *FEMS Microbiol. Ecol.* 92:fiv137. 10.1093/femsec/fiv137 26542074

[B32] Jaeggi T. Kortman G. A. Moretti D. Chassard C. Holding P. Dostal A. (2014). Iron fortification adversely affects the gut microbiome, increases pathogen abundance and induces intestinal inflammation in Kenyan infants. *Gut* 64 731–742. 10.1136/gutjnl-2014-307720 25143342

[B33] Janssen P. H. Kirs M. (2008). Structure of the archaeal community of the rumen. *Appl. Environ. Microbiol.* 74 3619–3625. 10.1128/AEM.02812-07 18424540 PMC2446570

[B34] Jin W. Cheng Y. F. Mao S. Y. Zhu W. Y. (2011). Isolation of natural cultures of anaerobic fungi and indigenously associated methanogens from herbivores and their bioconversion of lignocellulosic materials to methane. *Bioresour. Technol.* 102 7925–7931. 10.1016/j.biortech.2011.06.026 21719276

[B35] Jones E. Oliphant T. Peterson P. (2001). *Open Source Scientific Tools for Python.* Available at: http://www.scipy.org/

[B36] Kittelmann S. Naylor G. E. Koolaard J. P. Janssen P. H. (2012). A proposed taxonomy of anaerobic fungi (class *Neocallimastigomycetes*) suitable for large-scale sequence-based community structure analysis. *PLOS ONE* 7:e36866. 10.1371/journal.pone.0036866 22615827 PMC3353986

[B37] Koetschan C. Kittelmann S. Lu J. Al Halbouni D. Jarvis G. N. Müller T. (2014). Internal transcribed spacer 1 secondary structure analysis reveals a common core throughout the anaerobic fungi (Neocallimastigomycota). *PLOS ONE* 9:e91928. 10.1371/journal.pone.0091928 24663345 PMC3963862

[B38] Lazarevic V. Gaïa N. Girard M. François P. Schrenzel J. (2013). Comparison of DNA extraction methods in analysis of salivary bacterial communities. *PLOS ONE* 8:e67699. 10.1371/journal.pone.0067699 23844068 PMC3701005

[B39] Leimena M. Ramiro-Garcia J. Davids M. van den Bogert B. Smidt H. Smid E. (2013). A comprehensive metatranscriptome analysis pipeline and its validation using human small intestine microbiota datasets. *BMC Genomics* 14:530. 10.1186/1471-2164-14-530 23915218 PMC3750648

[B40] Li Y. D. Ma S. Zhang X. J. Huang S. W. Yang H. Zhao F. (2014). Evaluation of bacterial and archaeal diversity in the rumen of Xiangxi yellow cattle (*Bos taurus*) fed *Miscanthus sinensis* or common mixed feedstuff. *Ann. Microbiol.* 64 1385–1394. 10.1007/s13213-013-0783-x

[B41] Liggenstoffer A. S. Youssef N. H. Couger M. B. Elshahed M. S. (2010). Phylogenetic diversity and community structure of anaerobic gut fungi (phylum *Neocallimastigomycota*) in ruminant and non-ruminant herbivores. *ISME J.* 4 1225–1235. 10.1038/ismej.2010.49 20410935

[B42] Lu L. Xing D. Ren N. (2012). Bioreactor performance and quantitative analysis of methanogenic and bacterial community dynamics in microbial electrolysis cells during large temperature fluctuations. *Environ. Sci. Technol.* 46 6874–6881. 10.1021/es300860a 22612779

[B43] Mao S. Zhang M. Liu J. Zhu W. (2015). Characterising the bacterial microbiota across the gastrointestinal tracts of dairy cattle: membership and potential function. *Sci. Rep.* 5:16116. 10.1038/srep16116 26527325 PMC4630781

[B44] Marvin-Sikkema F. D. Richardson A. J. Stewart C. S. Gottschal J. C. Prins R. A. (1990). Influence of hydrogen-consuming bacteria on cellulose degradation by anaerobic fungi. *Appl. Environ. Microbiol.* 56 3793–3797. 2082826 10.1128/aem.56.12.3793-3797.1990 PMC185069

[B45] McCann J. C. Wickersham T. A. Loor J. J. (2014). High-throughput methods redefine the rumen microbiome and its relationship with nutrition and metabolism. *Bioinform. Biol. Insights* 8 109–125. 10.4137/bbi.s15389 24940050 PMC4055558

[B46] Meale S. J. McAllister T. A. Beauchemin K. A. Harstad O. M. Chaves A. V. (2012). Strategies to reduce greenhouse gases from ruminant livestock. *Acta Agric. Scand. A Anim. Sci.* 62 199–211. 10.1080/09064702.2013.770916

[B47] Orpin C. G. (1974). The rumen flagellate *Callimastix frontalis*: Does sequestration occur? *J. Gen. Microbiol.* 84 395–398. 10.1099/00221287-84-2-395 4448982

[B48] Orpin C. G. (1975). Studies on the rumen flagellate *Neocallimastix frontalis*. *J. Gen. Microbiol.* 91 249–262. 10.1099/00221287-91-2-249 1462

[B49] Orpin C. G. (1976). Studies on the rumen flagellate *Sphaeromonas communis*. *J. Gen. Microbiol.* 94 270–280. 10.1099/00221287-94-2-270 7636

[B50] Orpin C. G. (1977). The rumen flagellate *Piromonas communis*: its life-history and invasion of plant material in the rumen. *J. Gen. Microbiol.* 99 107–117. 10.1099/00221287-99-1-107 16983

[B51] Paul K. Nonoh J. O. Mikulski L. Brune A. (2012). *Methanoplasmatales*,” *Thermoplasmatales*-related archaea in termite guts and other environments, are the seventh order of methanogens. *Appl. Environ. Microbiol.* 78 8245–8253. 10.1128/AEM.02193-12 23001661 PMC3497382

[B52] Ramiro-Garcia J. Hermes G. D. A. Giatsis C. Sipkema D. Zoetendal E. G. Schaap P. J. (2016). NG-Tax, a highly accurate and validated pipeline for analysis of 16S rRNA amplicons from complex biomes. *F1000Res.* 5:1791 10.12688/f1000research.9227.1 PMC6419982 30918626

[B53] Russell J. B. Hespell R. B. (1981). Microbial rumen fermentation. *J. Dairy Sci.* 64 1153–1169. 10.3168/jds.S0022-0302(81)82694-X 7024344

[B54] Smilauer P. Leps J. (2014). *Multivariate Analysis of Ecological Data Using Canoco*, 2nd Edn. New York, NY: Cambridge University Press. 10.1017/CBO9781139627061

[B55] Suzuki M. T. Beja O. Taylor L. T. Delong E. F. (2001). Phylogenetic analysis of ribosomal RNA operons from uncultivated coastal marine bacterioplankton. *Environ. Microbiol.* 3 323–331. 10.1046/j.1462-2920.2001.00198.x 11422319

[B56] Suzuki M. T. Taylor L. T. DeLong E. F. (2000). Quantitative analysis of small-subunit rRNA genes in mixed microbial populations via 5′-nuclease assays. *Appl. Environ. Microbiol.* 66 4605–4614. 10.1128/AEM.66.11.4605-4614.2000 11055900 PMC92356

[B57] Ter Braak C. J. F. Smilauer P. (2012). *Canoco Reference Manual and User’s Guide: Software for Ordination (version 5.0).* Ithaca, NY: Microcomputer Power.

[B58] Teunissen M. J. Kets E. P. W. Op den Camp H. J. M. Huis in’t Veld J. H. J. Vogels G. D. (1992). Effect of coculture of anaerobic fungi isolated from ruminants and non-ruminants with methanogenic bacteria on cellulolytic and xylanolytic enzyme activities. *Arch. Microbiol.* 157 176–182. 10.1007/bf00245287 1550443

[B59] Van den Bogert B. De Vos W. M. Zoetendal E. G. Kleerebezem M. (2011). Microarray analysis and barcoded pyrosequencing provide consistent microbial profiles depending on the source of human intestinal samples. *Appl. Environ. Microbiol.* 77 2071–2080. 10.1128/AEM.02477-10 21257804 PMC3067328

[B60] Van den Bogert B. Erkus O. Boekhorst J. de Goffau M. Smid E. J. Zoetendal E. G. (2013). Diversity of human small intestinal *Streptococcus* and *Veillonella* populations. *FEMS Microbiol. Ecol.* 85 376–388. 10.1111/1574-6941.12127 23614882

[B61] Van Gastelen S. Antunes-Fernandes E. C. Hettinga K. A. Klop G. Alferink S. J. Hendriks W. H. (2015). Enteric methane production, rumen volatile fatty acid concentrations, and milk fatty acid composition in lactating Holstein-Friesian cows fed grass silage- or corn silage-based diets. *J. Dairy Sci.* 98 1915–1927. 10.3168/jds.2014-8552 25582590

[B62] Van Lingen H. Edwards J. Vaidya J. van Gastelen S. van den Bogert B. Saccenti E. (2017). Diurnal dynamics of gaseous and dissolved metabolites and microbiota composition in the bovine rumen. *Front. Microbiol.* 8:425. 10.3389/fmicb.2017.00425 28367142 PMC5355475

[B63] Van Lingen H. Plugge C. M. Fadel J. G. Kebreab E. Bannink A. Dijkstra J. (2016). Thermodynamic driving force of hydrogen on rumen microbial metabolism: a theoretical investigation. *PLOS ONE* 11:e0161362. 10.1371/journal.pone.0161362 27783615 PMC5081179

[B64] Villegas-Rivera G. Vargas-Cabrera Y. Gonzalez-Silva N. Aguilera-Garcia F. Gutierrez-Vazquez E. Bravo-Patino A. (2013). Evaluation of DNA extraction methods of rumen microbial populations. *World J. Microbiol. Biotechnol.* 29 301–307. 10.1007/s11274-012-1183-2 23054703

[B65] Wang Q. Garrity G. M. Tiedje J. M. Cole J. R. (2007). Naive Bayesian classifier for rapid assignment of rRNA sequences into the new bacterial taxonomy. *Appl. Environ. Microbiol.* 73 5261–5267. 10.1128/AEM.00062-07 17586664 PMC1950982

[B66] Yu Y. Lee C. Kim J. Hwang S. (2005). Group-specific primer and probe sets to detect methanogenic communities using quantitative real-time polymerase chain reaction. *Biotechnol. Bioeng.* 89 670–679. 10.1002/bit.20347 15696537

[B67] Yu Z. Morrison M. (2004). Improved extraction of PCR-quality community DNA from digesta and fecal samples. *Biotechniques* 36 808–812. 15152600 10.2144/04365ST04

[B68] Zhou M. Hernandez-Sanabria E. Guan L. L. (2009). Assessment of the microbial ecology of ruminal methanogens in cattle with different feed efficiencies. *Appl. Environ. Microbiol.* 75 6524–6533. 10.1128/AEM.02815-08 19717632 PMC2765141

[B69] Zoetendal E. G. Heilig H. G. Klaassens E. S. Booijink C. C. Kleerebezem M. Smidt H. (2006). Isolation of DNA from bacterial samples of the human gastrointestinal tract. *Nat. Protoc.* 1 870–873. 10.1038/nprot.2006.142 17406319

