# Supplementary material for: The Effect of DNA Extraction Methods on Observed Microbial Communities from Fibrous and Liquid Rumen Fractions of Dairy Cows
Source: Front Microbiol. 2018 Jan 31;9:92. doi: 10.3389/fmicb.2018.00092 (PMC5797766; doi:10.3389/fmicb.2018.00092)
Supplement: Supplementary file 1 [file Data_Sheet_1.pdf]

Supplementary Material

The effect of DNA extraction methods on observed microbial communities from fibrous and liquid rumen fractions of dairy cows

Jueeli D. Vaidya[1,2*], Bartholomeus van den Bogert[1,2,#], Joan Edwards[1,2], Jos Boekhorst[3], Sanne van Gastelen[1,4], Edoardo Saccenti[5], Caroline M. Plugge[2] and Hauke Smidt[2]

[1] Top Institute Food and Nutrition, Wageningen, the Netherlands

[2] Laboratory of Microbiology, Wageningen University and Research, Wageningen, the Netherlands

[3] NIZO food research BV, Ede, the Netherlands

[4] Animal Nutrition Group, Wageningen University and Research, Wageningen, the Netherlands

[5] Laboratory of Systems and Synthetic Biology, Wageningen University and Research, Wageningen, the Netherlands

[#] Current address: BaseClear B.V, Leiden, the Netherlands

*** Correspondence:**
Jueeli D. Vaidya
jueeli.vaidya@wur.nl

Figure S1

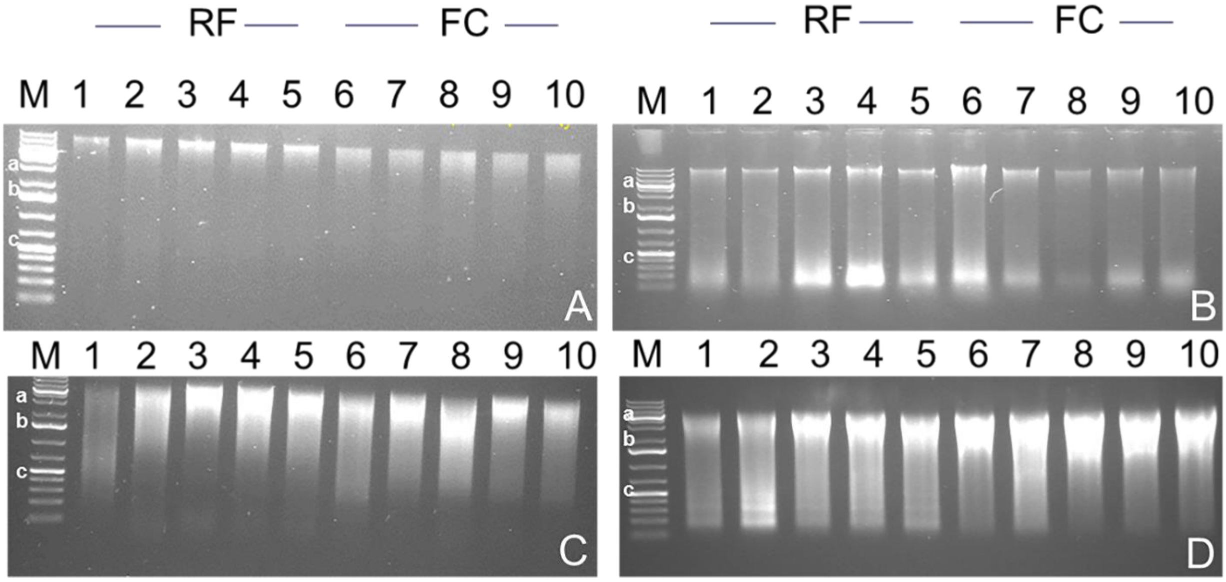

Figure S1 Integrity of genomic DNA extracted with different methods visualized on a 1% agarose gel. DNA obtained from rumen fluid (RF: lanes 1-5) and fibrous content (FC: 6-10) samples using method RBB (A), PBB (B), FDSS (C) and PQIAmini (D). Lanes represent cows fed the different diets as follows: MS100 (1 & 6), GS33MS67 (2 & 7), GS100 (3, 4, 8, and 9, as two technical replicates for this diet) and GS67MS33 (5 and 10). Lane M: 1 kb plus DNA size marker was used for all gels, a: 5000bp, b: 1000bp, c: 500bp.

Figure S2

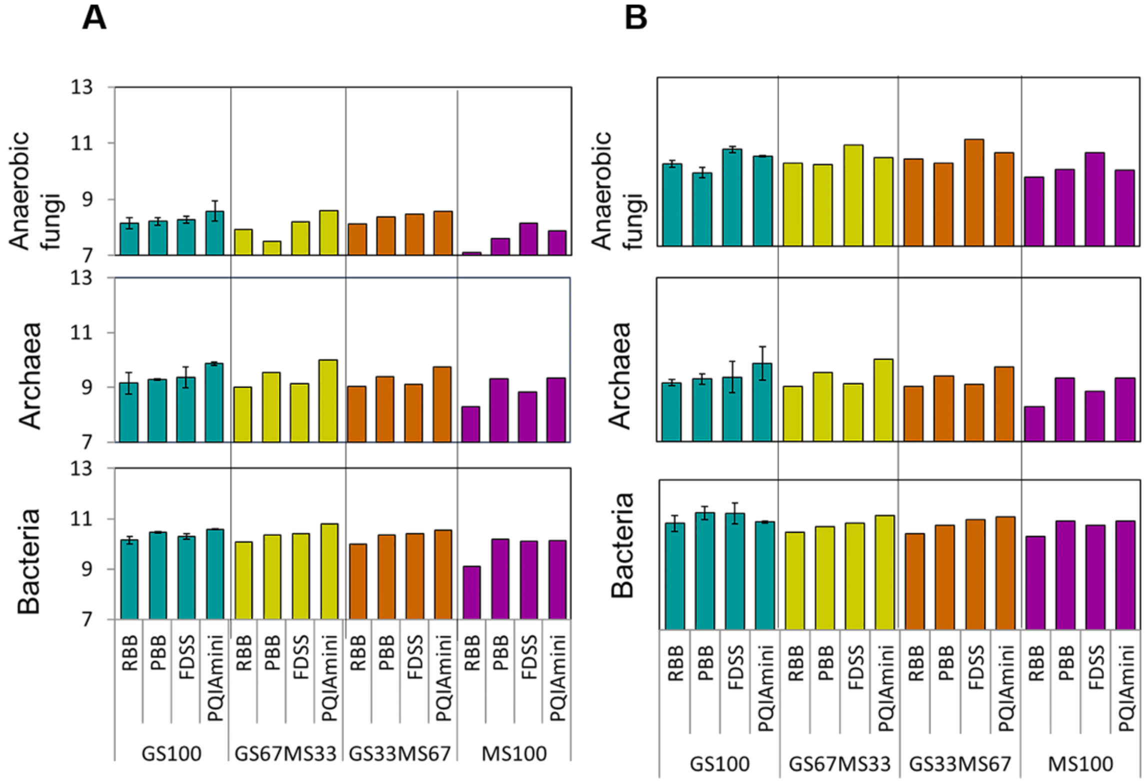

Figure S2. Bacterial and archaeal 16S rRNA and anaerobic fungal 5.8S rRNA gene copy numbers per ml of rumen fluid (RF) or gram fibrous content (FC) samples from dairy cows fed different ratios of grass silage (GS) to maize silage (MS), as measured in DNA extracted using four different methods (RBB, PBB, FDSS and PQIAmini). Bars represent the mean of triplicate qPCR determinations for a single sample DNA extract with the exception of GS100, where they represent the mean of duplicate DNA extracts and the error bars represent their standard deviation. Data is presented as log10 copy numbers per ml RF (A) and gram FC (B).

Figure S3

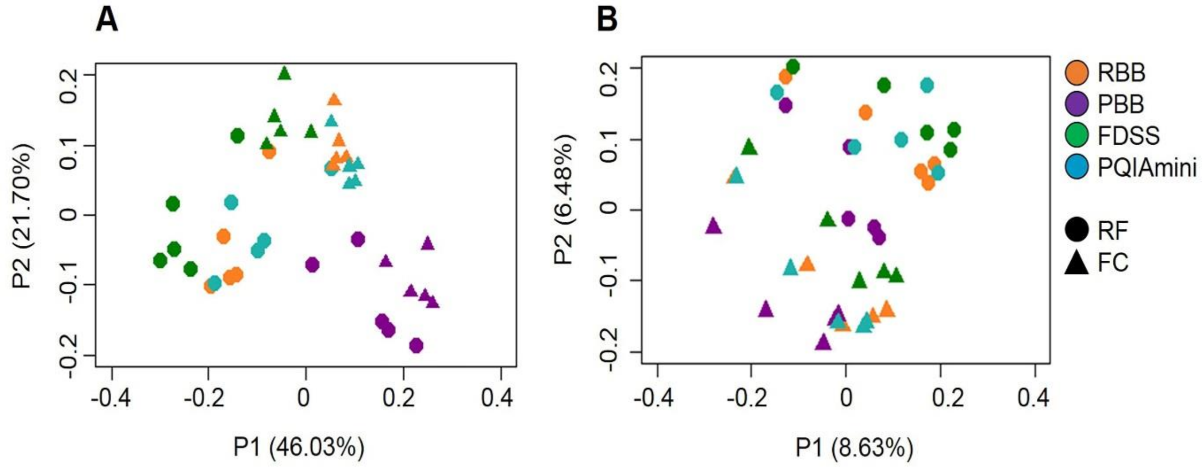

Figure S3. Weighted **(A)** and unweighted **(B)** UniFrac principal coordinate analysis (PCoA) of the rumen bacterial community at the OTU level. Datapoints are coded in terms of DNA extraction method (RBB, PBB, FDSS and PQIAmini) and fraction (rumen fluid (RF) and fibrous content (FC)). The GS100 diet has duplicate DNA extracts presented as individual datapoints. The percentages given at P1 and P2 indicate the amount of variation represented by the co-ordinate axes.

Table S1. Bacterial families and genera that significantly differ (p<0.05) in relative abundance in rumen fluid (RF) versus fibrous content (FC) fraction are shown in (A), and different DNA extraction method (RBB, PBB, FDSS and PQIAmini) comparisons are shown in (B). Relative abundance (%) values presented are the mean ± standard deviation.

A

Taxon	RF	FC	p value
**Family**			
Desulfobulbaceae	0.1 ± 0.1	0.0 ± 0.0	0.030
Fibrobacteraceae	0.6 ± 0.5	2.7 ± 2.3	0.020
Lachnospiraceae	8.0 ± 3.5	12.5 ± 3.3	0.006
Prevotellaceae	29.4 ± 11.1	11.6 ± 3.1	0.001
Ruminococcaceae	6.1 ± 1.7	9.0 ± 1.9	0.040
**Genus**			
Butyrivibrio	0.8 ± 0.3	1.7 ± 0.8	0.0001
Desulfobulbus	0.1 ± 0.0	0.0 ± 0.0	0.036
Fibrobacter	0.5 ± 0.4	2.8 ± 2.3	0.021
Oscillibacter	0.0 ± 0.0	0.1 ± 0.1	0.027
Paraeggerthella	0.1± 0.0	0.0 ± 0.0	0.0002
Prevotella	18.4 ± 8.1	4.7 ± 2.9	<0.0001
Pseudobutyrivibrio	0.2 ± 0.1	0.5 ± 0.2	0.010
Ruminococcus	0.3 ± 0.2	1.0 ± 0.5	<0.0001
Selenomonas	0.2 ± 0.1	0.0 ± 0.0	<0.0001
Succiniclasticum	2.1 ± 1.1	0.5 ± 0.3	<0.0001
Syntrophococcus	0.2 ± 0.1	0.3 ± 0.1	0.009

B

Taxon	DNA extraction method	p value
**Family**		
Anaerolineaceae	PQIAmini (0.1 ± 0.1) vs PBB (0.3 ± 0.1)	0.027
Anaerolineaceae	PBB (0.3 ± 0.1) vs RBB (0.1 ± 0.0)	0.038
Fibrobacteraceae	FDSS (3.2 ± 1.4) vs PBB (0.2 ± 0.1)	0.028
Fibrobacteraceae	PBB (0.2 ± 0.1) vs RBB (1.6 ± 0.7)	0.038
Halomonadaceae	FDSS (0.02 ± 0.0) vs PQIAmini (0.01 ± 0.01)	0.008
Halomonadaceae	PBB (0.2 ± 0.1) vs FDSS (0.02 ± 0.0)	0.028
Ruminococcaceae	FDSS (5.8 ± 0.6) vs PBB (9.6 ± 1.8)	0.038
**Genus**		
Fibrobacter	RBB (1.6 ± 1.4) vs PBB (0.1 ± 0.1)	0.038
Fibrobacter	PBB (0.1 ± 0.1) vs FDSS (3.5 ± 3.0)	0.038
